# Brain-specific functional relationship networks inform autism spectrum disorder gene prediction

**DOI:** 10.1038/s41398-018-0098-6

**Published:** 2018-03-06

**Authors:** Marlena Duda, Hongjiu Zhang, Hong-Dong Li, Dennis P. Wall, Margit Burmeister, Yuanfang Guan

**Affiliations:** 1 Department of Computational Medicine and Bioinformatics, University of Michigan, Ann Arbor, MI USA; 2 Center for Bioinformatics, School of Information Science and Engineering, Central South University, Changsha, China; 3 Department of Pediatrics, Division of Systems Medicine, Stanford University, Stanford, CA USA; 4 Department of Biomedical Data Science, Stanford University, Stanford, CA USA; 5 Molecular and Behavioral Neuroscience Institute, University of Michigan, Ann Arbor, MI USA; 6 Department of Human Genetics, University of Michigan, Ann Arbor, MI USA; 7 Department of Psychiatry, University of Michigan, Ann Arbor, MI USA; 8 Department of Internal Medicine, Usniversity of Michigan, Ann Arbor, MI USA; 9 Department of Electrical Engineering and Computer Science, University of Michigan, Ann Arbor, MI USA

## Abstract

Autism spectrum disorder (ASD) is a neuropsychiatric disorder with strong evidence of genetic contribution, and increased research efforts have resulted in an ever-growing list of ASD candidate genes. However, only a fraction of the hundreds of nominated ASD-related genes have identified de novo or transmitted loss of function (LOF) mutations that can be directly attributed to the disorder. For this reason, a means of prioritizing candidate genes for ASD would help filter out false-positive results and allow researchers to focus on genes that are more likely to be causative. Here we constructed a machine learning model by leveraging a brain-specific functional relationship network (FRN) of genes to produce a genome-wide ranking of ASD risk genes. We rigorously validated our gene ranking using results from two independent sequencing experiments, together representing over 5000 simplex and multiplex ASD families. Finally, through functional enrichment analysis on our highly prioritized candidate gene network, we identified a small number of pathways that are key in early neural development, providing further support for their potential role in ASD.

## Introduction

The term autism spectrum disorder (ASD) describes a range of complex neurodevelopmental phenotypes marked by impaired social interaction and communication skills along with restricted interests and repetitive behaviors^1^. Incidence rates of ASD have been steadily increasing in recent years, currently estimated to affect >1% of the population worldwide^2,3^. High rates of concordance observed in studies of monozygotic twins^4,5^ and well-characterized monogenic manifestations of autism (known as syndromic autism) provide support for a genetic contribution for the disorder. Recently, a number of large-scale family-based sequencing studies^6–13^ have been conducted, aimed at uncovering potentially causal genomic variants for ASD. These studies, which primarily focus on de novo loss-of-function (LOF) mutations, have found significant associations with autism in anywhere from 65 to 150 genes; however, statistical genetic models estimate the total number of genes that contribute to ASD at ~500^14^. Uncovering the remaining contributory genes in ASD will provide new insights into the molecular etiology of the disorder, as well as identify novel targets for pharmaceutical intervention.

The complexity of ASD points to the disregulation of not one, but multiple pathways and biological processes. Network studies have proven useful in refining our understanding of the molecular basis of ASD^15–25^; however, many of these analyses are limited by networks built upon functional associations in a single context or a lack of tissue specificity. Studying known ASD genes in the context of a tissue-specific functional network built on a variety of integrated data types could provide a means of elucidating novel candidate genes involved in autism susceptibility based on the biological pathways in which they are involved.

A recent work^26^ addressed some of these shortcomings to predict a genome-wide ranking of ASD-related genes using an integrated genomic network based on a variety of tissue-specific functional data types. Our study builds upon this previous work in three ways. First, we use a Bayesian model to build a functional relationship network that not only integrates brain-specific gene expression data with various protein-protein interaction data, but does so across three different species (human, mouse, and rat), providing a more complete molecular foundation upon which to build our network. Second, we utilize a more refined truth set of ASD genes (143 vs 594), including genes known to cause syndromic autism, to train our model. Third, we utilize a sophisticated random forest ensemble model to generate our ASD gene ranking, which is better suited to this prediction task than simple linear or kernel-based models. The random forest model was trained on the genome-wide functional connections between our high-confidence ASD genes and identifies genes with similar connectivity patterns within the network as likely autism candidate genes. Our methods constitute a novel algorithm with enhanced ability to discriminate known ASD genes from control genes and to prioritize genes with observed *de novo* mutations in autism probands. The resulting genome-wide ranking of ASD genes can be used to prioritize candidate genes identified through sequencing studies and to uncover possible pathways that may be implicated in autism. Moreover, our methods can be adapted for candidate gene discovery in other complex disorders.

## Methods

### Gene set curation

To compile our truth set of known ASD genes, we combined high confidence genes from the SFARI Gene database^27^ (http://gene.sfari.org) with a list of 65 genes recently reported by Sanders et al.^7^. The SFARI Gene database contains a manually annotated list of over 600 genes along with citations supporting their association with ASD. These genes are categorized based on the strength of the evidence linking each gene with autism. To ensure our truth set of known ASD genes contained only high fidelity genes, we included genes in the SFARI categories 1 (high confidence) and 2 (strong candidate) (*n* = 55), which require stringent inclusion criteria regarding statistical significance and replication of findings. In addition to the category 1 and 2 genes, we also included SFARI syndromic genes in our ASD truth set (*n* = 79). Considering the highly penetrant nature of many of these syndromic mutations, we intuited that patterns in the functional relationships between these genes and their neighbors would be highly valuable in the prediction of novel candidate genes for idiopathic ASD as well. 18 genes in the syndromic category overlapped with genes in the category 1 and 2 lists. Additionally, we considered a list of genes (*n* = 65) proposed by Sanders et al. as part of our ASD truth set. These genes are the result of a rigorous de novo mutation analysis across exomes of individuals from the Autism Genome Project (AGP), Simons Simplex Collection (SSC), and Autism Sequencing Consortium (ASC), which controlled for the confounder of intellectual disability. From the Sanders list, 37 of the 65 genes overlapped with genes in the SFARI category 1 and 2 list and 13 overlapped with genes in the SFARI syndromic list. Overall, our ASD truth set comprised 143 unique, high confidence autism-associated genes. Training a valid machine learning model requires examples from both the positive (known ASD) and negative (known non-ASD) class. However, selection of “true” negative class examples for training in this context is less straightforward than selection of positive class examples. To enable a more reliable comparison between works, we elected to use the non-ASD gene list curated by Krishnan et al.^26^. According to their report, Krishnan et al. identified 1189 genes associated with non-mental health diseases, as annotated in OMIM. Upon investigation of this gene set, we found 13 of these 1189 “non-ASD” genes overlapped with genes in our ASD truth set—11 genes from the SFARI syndromic gene list (*BRAF, CACNA1C, CDKL5, CHD7, DMD, GATM, KCNJ10, NIPBL, OCRL, PTPN11*, and *SGSH*) and two genes from the Sanders list (*AKAP9* and *TCF7L2*). After removing these, we were left with 1176 genes in our non-ASD training set.

### Cross-species brain-specific functional relationship network

We built a cross-species brain-specific functional relationship network (FRN) of over 20,000 genes through Bayesian network integration of a diverse set of functional genomic data types derived from human, mouse (*Mus musculus*) and rat (*Rattus norvegicus*) experiments. Combining molecular information across species gives rise to a more complete functional network by providing information for tissue types for which human samples are rare. The four data types included in network construction were as follows.

#### Microarray

Gene Expression Omnibus (GEO)^28^ is a repository for microarray data sets, measuring gene expression across a variety of conditions, tissue types and species. From GEO, we manually identified a subset of non-cancer-related expression datasets in brain tissue from human, rat, and mouse (*n* = 213) based on the annotations provided. Expression data were passed through a pipeline that included log_2_ transformation of probe set expression values, discarding probe sets with missing data for >30% of subjects and imputing any missing expression values using a *k*-Nearest Neighbor method for the remaining probe sets, and computing gene expression values by averaging the expression values for all probe sets mapped to each gene. For all possible pairs of genes, the Fischer *z*-transformed Pearson correlation was computed and used as input for network generation.

#### Protein–protein interactions

Protein–protein interaction (PPI) data were obtained from the Biomolecular Interaction Network Database (BIND)^29^, the Biological General Repository for Interaction Datasets (BioGRID)^30^, IntAct^31^, the Molecular Interaction database (MINT)^32^, and the Munich Information Center for Protein Sequences (MIPS)^33^. Redundant interactions were removed, resulting in a total of 30,800 PPIs informing the FRN.

#### Protein docking

We calculated a quantitative physical interaction (PI) score for all protein isoform pairs in mouse. For each gene pair, this PI score was calculated as the maximum of all its isoform pairs, per the SPRING algorithm^34^. Each mouse gene was mapped to its homologous human gene, from which we obtained PI scores for 5,064,860 gene pairs.

#### Phenotype annotations

Using the basic intuition that genes with similar phenotypic landscapes likely have underlying functional relationships, we utilized phenotype annotations from the Mouse Genome Informatics (MGI) database. In these data, each gene was annotated to one or more broad phenotypes, such as mortality/aging or behavior. For every possible pair of genes, we utilized the number of overlapped phenotypes as a feature for FRN construction.

The integration of these diverse data types provides valuable insights into different aspects of functional relationships between genes that cannot be fully captured by a single experimental method, but is accompanied by the caveat of the varying reliability between datasets. By assigning weights to each dataset, we can exploit the unique information provided from each source while preventing potentially spurious results from biasing the composition of the entire network. We utilized a two-layer Bayesian network to perform weighted integration of our four data types and provide the final probability of functional interaction between all pairs of 21,122 genes. This posterior probability of functional interaction was calculated for each gene pair with the formula:

1 $$P\left( {{\mathrm {FR}}{\mathrm{|}}E_1,E_2,E_3, \ldots ,E_n} \right) = \frac{1}{C}P\left( {\mathrm {FR}} \right)\mathop {\prod}\nolimits_{i = 1}^n {P\left( {E_i{\mathrm{|}}{\mathrm {FR}}} \right)}$$

where FR represents a functional relationship, *E*_*i*_ represents the *i*th piece of evidence, i.e., the score of the pair in each dataset *i*, and *C* is a scaling factor for normalization. Intuitively, this probability *P*(FR_*i,j*_) for two genes *i* and *j* translates to the likelihood, given existing data as well as the accuracy and coverage of each input dataset, that genes *i* and *j* are involved in the same biological process.

To determine the weights assigned to each dataset, we assembled a gold standard list of true functional gene relationships from Gene Ontology (GO)^35^ biological processes and Kyoto Encyclopedia of Genes and Genomes (KEGG)^36^ pathways. We limited this gold standard list to include only those pathways/GO terms with 5–300 associated genes to avoid extremely specific or exceedingly broad pathways that would be uninformative in this context. Using these criteria, we identified functional gene relationships in human, mouse and rat resulting in 778,312, 667,165, and 460,514 functionally related gene pairs, respectively. Rat and mouse genes were mapped to human homologs, creating a final gold standard list of 1,016,901 related gene pairs.

Our data integration method consisted of a two-layer Bayesian model. In the first layer, brain-specific microarray datasets from GEO were consolidated into a combined expression score using Eq. (1). In the second layer, PPI, protein docking and functional annotations were integrated with the combined expression score from the first layer, also using Eq. (1). The result was a square matrix (*n* = 21,122) where each entry *i*,*j* was the probability *P*(FR_*i,j*_) defined above, representing the edge weights in our FRN. The network construction method presented here is an iterative improvement on analogous methods used for construction of other functional molecular networks in previous works^37,38^.

### Network-based classifier training and whole-genome prediction

We utilized a machine learning approach to distinguish between ASD-related and non-ASD-related genes. As stated above, our training set consisted of 1319 genes: 143 known ASD genes and 1176 genes with no evidence of ASD association. Rows from the FRN matrix that corresponded to these labeled genes were used to train the classifiers.

We trained and optimized five different machine learning models: support vector machine (SVM) with linear kernel, random forest, extremely randomized trees, bagging ensemble of random forests, and AdaBoost ensemble of random forests. Our training approach consisted of a stratified five-fold cross validation (CV) with a nested grid-search for parameter optimization and class weight parameters to offset the imbalance in class size. Performance of each machine learning model was estimated on the hold-out set for each fold of the CV using receiver-operating characteristic area under the curve (ROC-AUC). Based on the average CV performance, we chose the random forest as our final model for further prediction. Model training was performed in Python using the package scikit-learn^39^.

Genome-wide prediction of ASD association was done in one of two ways, as proposed by Krishnan et al.^26^. For initially “unlabeled” genes (i.e., genes not included in the 1319 genes used in training), prediction probabilities were recorded over all five folds of CV and averaged to determine the final predicted probability of ASD association. For each of the 1319 “labeled” genes, prediction probability was recorded only for the CV fold in which the gene was included in the hold-out set, which became the final prediction probability assigned to that gene. Finally, we constructed a genome-wide ranking in terms of ASD association by sorting all 21,112 genes by their ASD association probabilities.

### Validation in independent sequencing studies

We utilized results from two independent DNA sequencing experiments^10,13^ comprising a total of 4583 ASD families to evaluate the validity of our gene ranking. In the results from both studies, we focused on genes harboring de novo loss-of-function (LOF) mutations.

In 2014, Iossifov et al. conducted an exome-sequencing analysis of 2517 families in the SSC^10,40^. This study identified 350 LOF de novo mutations in the ASD probands (27 of which were observed in more than one proband, i.e., “recurrent”) and 174 LOF de novo mutations in the unaffected siblings. We observed the distribution of genes in each of these three gene lists across our entire gene ranking. Our gene ranking was first split into 10 evenly binned deciles, and using these bins we computed a decile enrichment test^26^. For each of the three gene lists, we tested whether a larger proportion of the genes were observed in the first decile than expected using a binomial test. The expected proportion (0.157) was determined using the distribution of genes with synonymous de novo mutations in unaffected siblings (*n* = 440) across our decile ranking.

Considering the publication of the SSC results preceded the aggregation of data for our ASD truth set, it is possible that these resulting genes (specifically the 27 recurrent proband LOF genes) were considered in the manual curation of the SFARI category 1 and 2 genes and were therefore included in part of our training set. To ensure a robust evaluation of our gene ranking, we performed an analogous analysis using the results of a recently published whole-genome sequencing study of the MSSNG cohort, which were unpublished at the time of our data collection^13^. This analysis consisted of WGS data from over 5000 samples (including 2626 ASD samples) in families independent of the SSC. In total, this analysis identified 214 de novo LOF mutations in probands and 61 statistically significant de novo LOF or missense mutations, 18 of which have been previously unreported in terms of ASD association. We performed decile enrichment on these three gene sets (total LOF, statistically significant LOF, previously unreported significant LOF) as described above, using the same expected proportion of top-decile genes identified from SSC sibling control synonymous mutation distribution.

#### Comparison against other neurological disease genes

To ensure our genome-wide ranking was enriched for true ASD-related genes, and not simply inflated by genes that are integral to overall cognition and brain function, we performed decile enrichment as described above on genes known to be related to Alzheimer’s disease (AD) and Parkinson’s disease (PD)—neurological disorders that are unrelated to autism, as well as ataxia—a neurological condition with known ASD comorbidity. We identified 20 genes known to be implicated in AD^41^, 22 in PD (http://www.pdgene.org/top_results), and 51 in ataxia (manual expert curation using GeneCards), again using the sibling synonymous mutation distribution as a benchmark for expected top-decile enrichment.

### Functional network characterization

We sought to identify and characterize functional clusters within our predicted autism genes. We began with the network produced by our top decile genes and their direct connections in our FRN (connections are defined by FRN probability score ≥0.98). We then used the GLay^42^ implementation of the Girvan–Newman fast greedy algorithm^43^ to perform community clustering within the ASD-associated network. This algorithm identifies stable community structure within large networks by iteratively removing edges with the highest “betweenness”—a metric that produces higher values for edges between communities rather than those within communities.

We performed functional enrichment testing to identify GO biological processes that were significantly enriched in identified clusters. False discovery rate correction was performed using the Benjamini–Hochberg method, and GO biological processes whose Q-value ≤0.01 were considered significantly enriched.

## Results

Using a machine learning approach, we developed a genome-wide prediction for ASD-related genes based on a cross-species, brain tissue-specific functional genomic relationship network. We evaluated these candidate gene predictions using results from two independent next-generation sequencing experiments and identified several functional sub-clusters of interacting genes that may shed new light on the molecular etiology of autism.

### Prioritization of ASD candidate genes

We developed a functional relationship network of 21,112 genes by integrating publicly available tissue-specific microarray, protein interaction, and phenotype annotation datasets from human, mouse, and rat. We implemented a two-layer Bayesian framework to weight and combine evidence from each dataset individually, resulting in a probabilistic functional interaction score for all pairs of genes. This edge-weight matrix became the feature basis for our machine learning classification system.

**Fig. 1:**
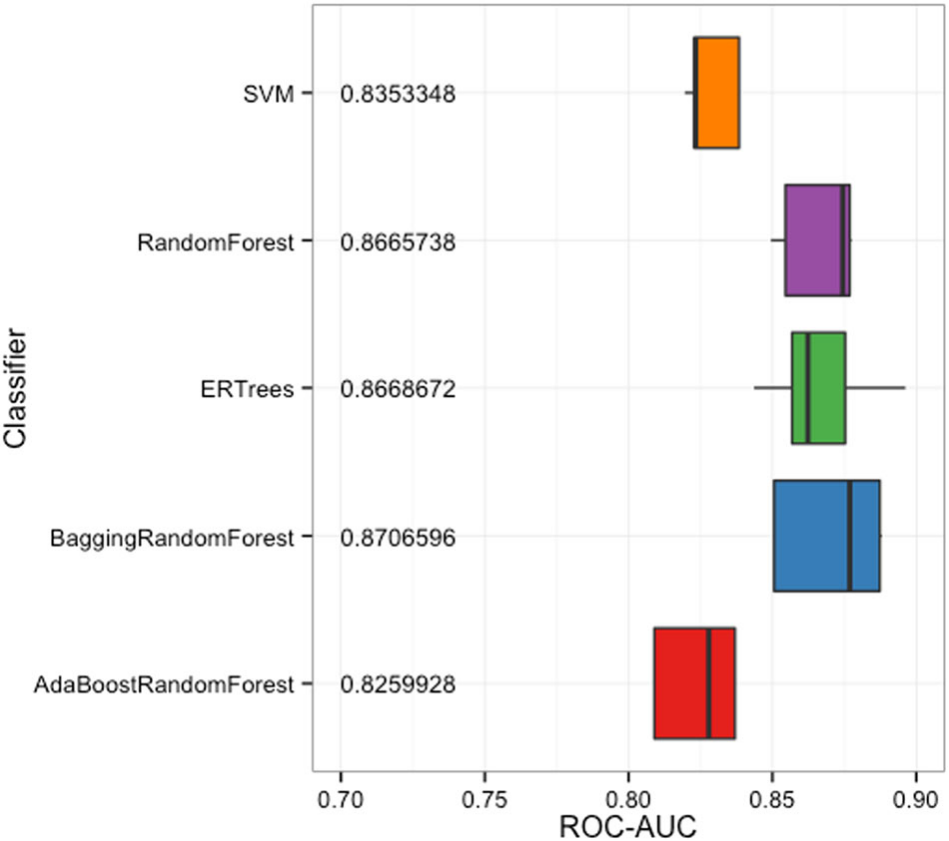
**ROC-AUC for all classifiers over five-fold cross validation**

To generate the most accurate predictions for ASD-related genes, we curated a highly specific list of 143 genes known to be implicated in autism. These genes were extracted from the SFARI Gene database^27^ gene lists from categories 1 (high confidence), 2 (strong candidate), and S (syndromic). We augmented this SFARI-based gene list with statistically significant genes identified in a recent exome sequencing study by Sanders et al.^7^ to finalize our ASD-truth gene set. Using the control gene set proposed by Krishnan et al.^26^, we trained five independent machine learning models to distinguish between autism and non-autism genes. Classifier training was implemented with a five-fold cross-validation (CV) scheme, with a nested grid-search CV for parameter optimization. Based on the performance of each model over the five-fold CV, we chose the random forest as our final model (ROC-AUC = 0.875 ± 0.024) (Fig. 1). The performance of this random forest model was stable over 10 rounds of re-seeded five-fold CV (Supplementary Fig. 1). Using this model, genome-wide probability of ASD association was calculated as the prediction value in the CV fold for which that gene was in the hold-out set for all labeled genes in the training set, or as the average prediction value across all five folds of CV for all remaining unlabeled genes (Supplementary Table 1).

**Fig. 2:**
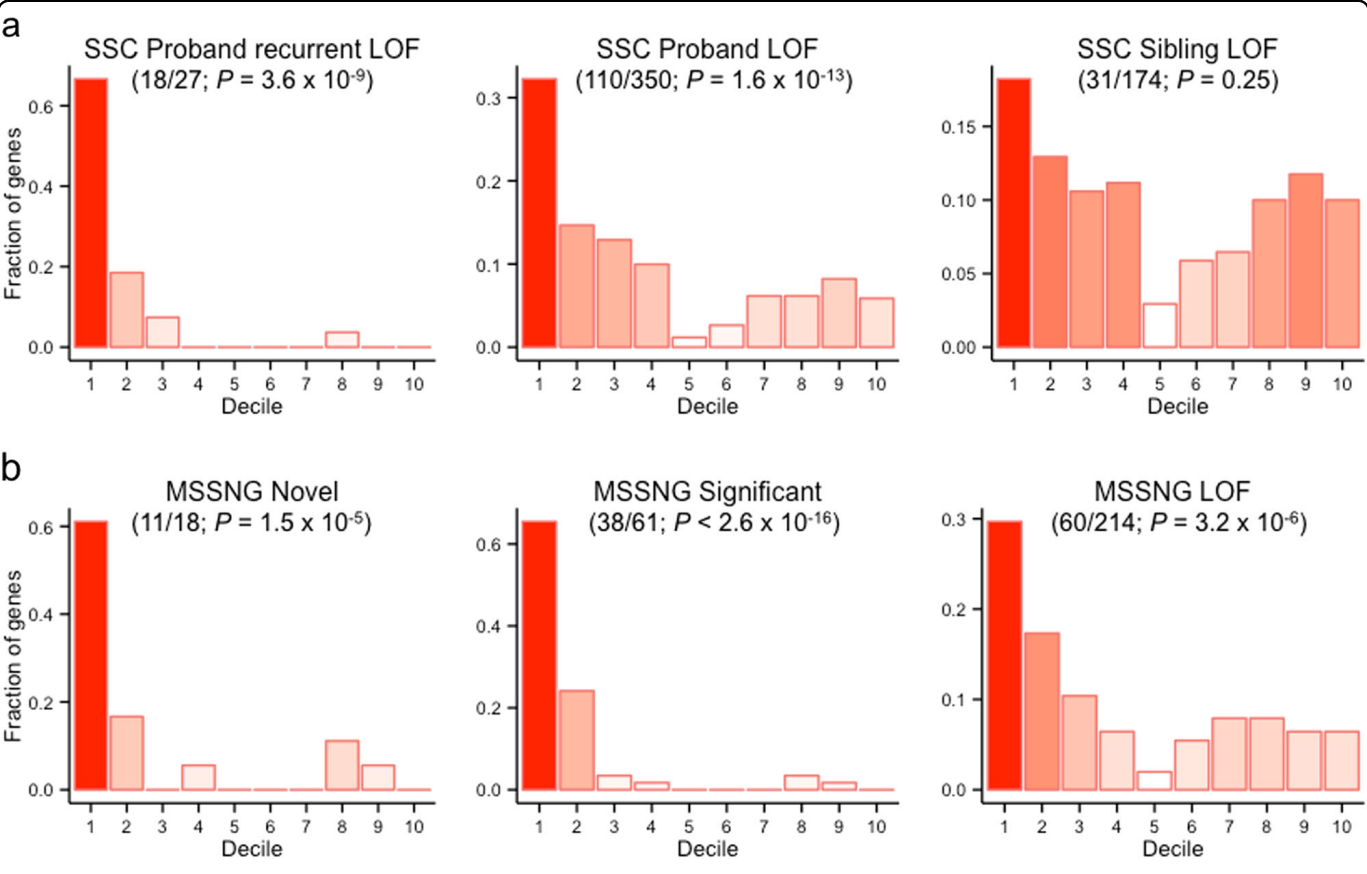
**Decile enrichment of ASD-related genes from two independent cohorts.** Distribution of genes with de novo LOF mutations in probands in SSC (**a**) and MSSNG (**b**) cohorts across our predicted gene ranking

We evaluated our ASD gene predictions using results from two independent sequencing studies, focusing on identified de novo LOF mutations. The first, an exome-sequencing study of 2517 families in the SSC^10^, identified 350 genes with de novo LOF mutations in probands (27 recurrent, or present in more than one proband) and 174 genes with de novo LOF mutations in unaffected siblings. We found significant enrichment of proband de novo LOF mutations (binomial test; *P* = 1.6 × 10^−13^) and recurrent proband de novo LOF mutations (*P* = 3.6 × 10^−9^) in our top decile genes, identifying as many as 67% of these genes in the top 10% of our ranking. There was, however, no significant enrichment of the genes harboring de novo LOF mutations in the unaffected siblings among our gene ranking (*P* = 0.25; Fig. 2a). Furthermore, we validated our ranking against results from a whole-genome sequencing experiment in the MSSNG cohort, a set of over 5000 samples independent from the SSC, that was unpublished at the time of our ASD gene set curation and model training^13^. This study identified 214 total genes with de novo LOF mutations in probands and 61 genes that reached genome-wide significance for association with ASD, 18 of which had no prior evidence of association with ASD in the literature. Again, we found significant enrichment of these gene lists in the top decile of our ranking (binomial test; *P* = 3.2 × 10^−6^, <2.6 × 10^−16^, 1.5 × 10^−5^, respectively; Fig. 2b).

**Fig. 3:**
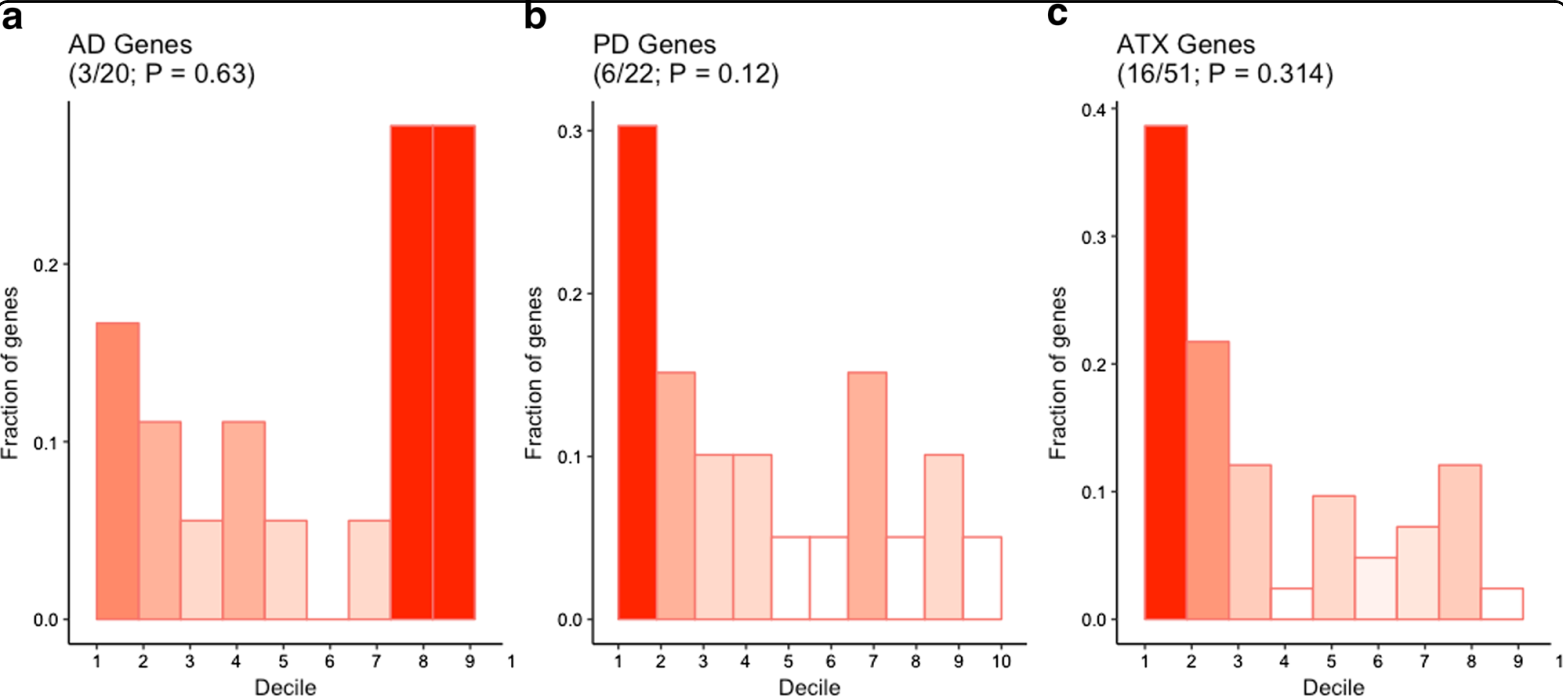
**Absence of decile enrichment of comorbidly-related genes.** Distribution of genes known to be associated with Alzheimer disease (AD) (**a**), Parkinson’s disease (PD) (**b**), and ataxia (ATX) (**c**) across our predicted gene ranking

While our gene ranking showed high sensitivity (i.e., the ability to correctly prioritize ASD genes), we wanted to also test the specificity, or the ability to downweight non-autism genes, to ensure we were not simply observing an enrichment for genes involved in basal brain function. To understand the specificity of our ranking, we tested the distribution of genes known to be implicated in AD, PD, and ataxia across our ASD-based ranking. We found no significant enrichment of the AD (binomial test; *P* = 0.63), PD (*P* = 0.12), or ataxia (*P* = 0.31) gene sets in the top decile of our ranking (Fig. 3), supporting the indication that our ranking is enriched for ASD-specific genes and not just for neural function.

### Characterization of ASD brain network

To better understand the molecular basis of autism, it is necessary not only to identify and prioritize candidate genes, but also to study the interactions and the shared biological processes in which those genes participate. We defined the ASD brain network as the connections between our top decile (*n* = 2111) genes and their immediate neighbors within our cross-species brain-specific network. Following the intuition that functionally related genes tend to share many connections and develop local “neighborhoods” within a larger network, we sought to identify such functional neighborhoods using the GLay^42^ community finding algorithm.

**Fig. 4:**
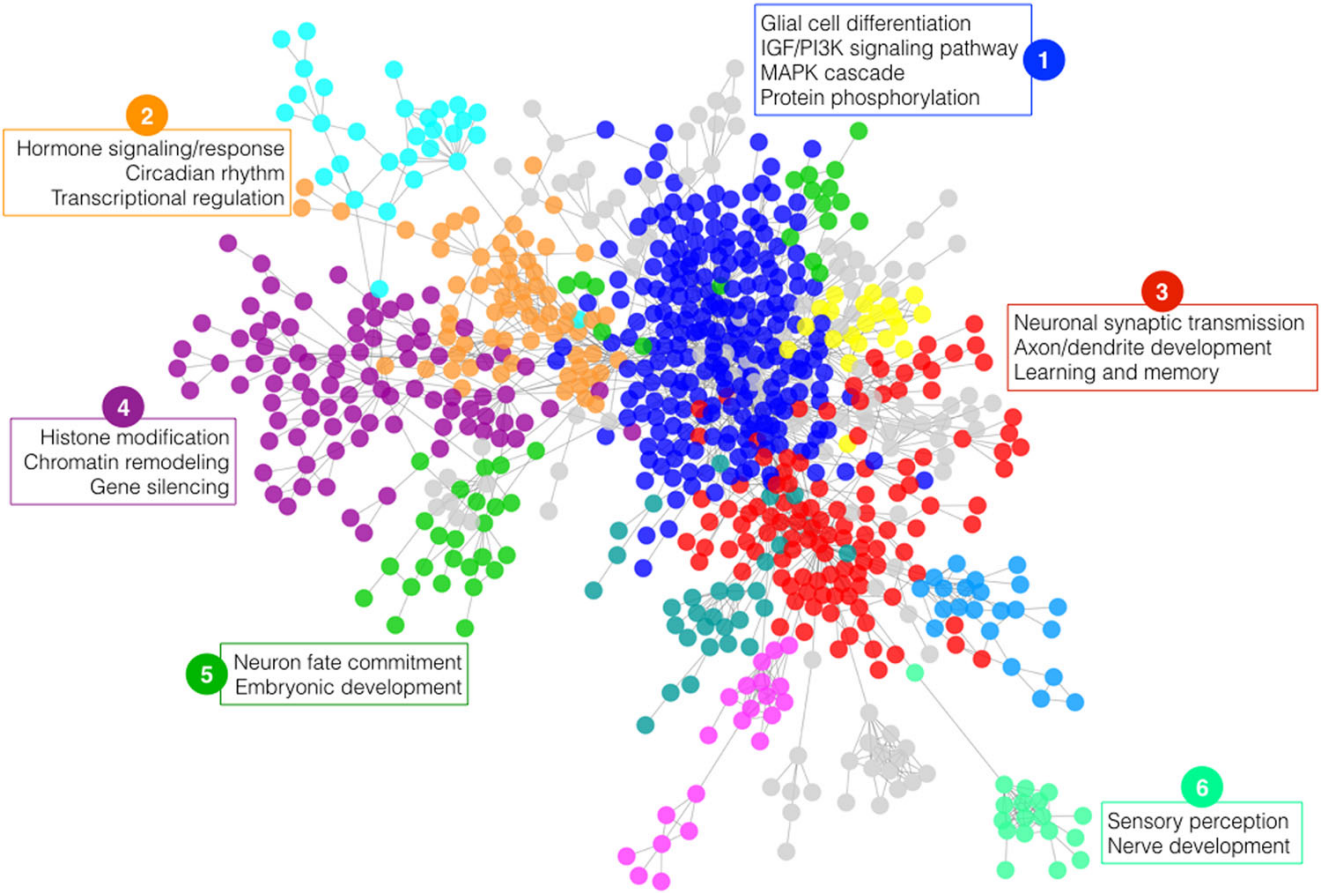
**Functional clusters in the ASD brain network**

Biological process enrichment testing revealed that the ASD brain network organized into a number of functionally distinct clusters, each having relevant associations with the autism phenotype (Fig. 4). Cluster 1 showed enrichment for several signaling pathways with evidence of involvement in ASD, including IGF/PI3K^44^, canonical Wnt^45^, and MAPK cascade^46^. Biological processes such as chromatin remodeling, histone modification, and transcriptional regulation that have been previously implicated in ASD are captured in clusters 2 and 4^12^. Furthermore, a number of developmental processes are enriched in our clusters, including embryonic development and neuron fate commitment in cluster 5 and nervous system development in cluster 6. Enrichment of axonal and dendritic development and morphogenesis in cluster 3 is of particular interest considering the evidence of increased dendritic spine density in ASD^47,48^.

## Discussion

The genetic basis of autism is as complex as its phenotypic presentation is variable, posing numerous challenges in the identification and characterization of the full complement of ASD risk genes. Continuously growing sample sizes of ASD-related high-throughput sequencing studies provide valuable resources for identification of strongly associated mutations, however even these large-scale studies may overlook potentially contributory mutations with small to moderate effect sizes. Novel methods to nominate potential ASD susceptibility genes within their biological context are needed to fully uncover the molecular underpinnings of the disorder.

Here we present a computational approach for identification of novel ASD candidate genes using a cross-species brain-specific integrated functional relationship network and advanced machine learning techniques. Our method improves upon previous work in a number of ways. Firstly, the underlying gene network integrated a broad range of functional genomic data from three different species (human, mouse, and rat). Using microarray data from homologous genes in mouse and rat provides valuable insights into gene expression in tissue types for which human samples may be relatively rare, as is the case with brain tissue. Concatenating these results with those from human-based studies within a Bayesian framework enables a more robust representation of true functional relationships between genes within our FRN. Secondly, our selection criteria for curating our training gene sets was more stringent than those used in Krishnan et al^26^. resulting in a true ASD gene set more than 75% smaller than their true ASD gene set even after inclusion of syndromic ASD genes, which were omitted from their analyses.

The implication of this difference is two-fold. First, by limiting our training set to bona fide autism genes, we ensure that our classifier learns connectivity patterns between genes with strong, repeated evidence of association with ASD and decreases the likelihood of erroneously prioritizing genes with no true molecular connection to the disorder. Second, excluding syndromic ASD genes from the truth set (as was done in previous work) omits genes in which the autism phenotype is highly penetrant, disregarding a significant portion of the biological context they are attempting to leverage. In fact, 11 of the syndromic ASD genes (14%) were included in the Krishnan et al. control gene set, which likely contributed significantly to the variation in results between our studies. Finally, we utilize a random forest model to construct our ASD classifier, as opposed to the linear SVM used in previous work. Intuitively, the random forest is better suited to this classification task than linear SVM, as it does not expect linear relationships between features and is more robust to a high-dimensional feature space. Furthermore, our random forest implementation includes an embedded feature selection step, where the maximum number of features used in construction of the ensemble is the square root of the total feature set, or in this case 145 features. By definition SVM constructs a linear boundary between all features in the feature set, which can lead to overfitting when the feature set is significantly larger than the number of training samples, as is the case here and in Krishnan et al. Even more sophisticated SVM implementations, such as polynomial kernel, are prone to overfitting in this context, and therefore were excluded from our study to bypass the computationally expensive step of optimally tuning a huge parameter space. Together, these enhancements culminate in significantly improved performance, including an increase in our classifier’s ability to discriminate between known ASD and control genes (ROC-AUC = 0.87 vs. 0.80), as well as an increase in the number of genes with de novo LOF mutations observed in actual ASD probands that were highly prioritized in our ranking (31.4% vs. 20.8%).

Our methods not only predicted genes with clear evidence of ASD association but also proposed a number of novel genes with high probability of contribution to autism risk. Community clustering and functional enrichment testing of these highly prioritized genes revealed several functional clusters consistent with those previously reported in ASD literature. For instance, cluster 3 includes known autism genes such as *BRAF*, *PTEN*, and *NTRK1* involved in synaptic transmission and learning/memory^47,48^, and ASD genes *CHD7, CHD8*, and *CTCF* known to be involved in chromatin remodeling and histone modification are found in cluster 4^12^. Furthermore, increasing evidence has implicated circadian rhythms^49,50^ and MAPK signaling pathways^46,51^ as important biological processes contributing to ASD, which were represented in clusters 1 and 2. The resulting functional clusters indicate that our highly prioritized ASD genes are indeed biologically meaningful to the autism phenotype. This clustering can also provide biological insights into the functional roles of highly ranked genes that are currently uncharacterized or poorly understood, making strides toward closing the gap between genotype and phenotype in this complex disorder.

Overall, our probabilistic whole genome ranking serves as a valuable resource for ASD gene discovery endeavors, enabling researchers to better frame results from sequencing experiments and identify promising candidate genes for further exploration. Moreover, our approach facilitates identification of genes with potentially small or moderate effect sizes that may not reach significance in large-scale sequencing studies by prioritizing genes with connectivity patterns matching those of known ASD genes, and are therefore likely to participate in similar biological pathways. Additionally, our data-driven approach can easily be utilized to predict genes with potential pathogenic involvement in a number of complex brain disorders.

### Code availability

Code used to generate results is available at https://github.com/GuanLab/ASD_FRN.

## Electronic supplementary material

Supplemental Figure 1

Supplemental Table 1

Supplemental Table 2
