# Supplementary figures and images for: Brain-specific functional relationship networks inform autism spectrum disorder gene prediction
Source: Transl Psychiatry. 2018 Mar 6;8:56. doi: 10.1038/s41398-018-0098-6 (PMC5838237; doi:10.1038/s41398-018-0098-6)

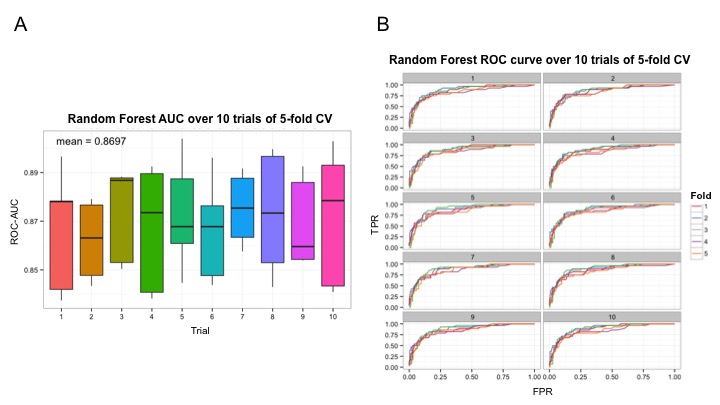

Supplement: Supplementary file 1 — Supplemental Figure 1 [file 41398_2018_98_MOESM1_ESM.jpg]
